# Ultrasound-Assisted Pocket Creation in Subglandular Breast Augmentation

**DOI:** 10.1093/asjof/ojag016

**Published:** 2026-01-28

**Authors:** Héctor Cesar Durán Vega, Ana Sofia Magallanes, Raúl Manzaneda Cipriani, Emanuel Flores

## Abstract

Electrocautery remains the conventional technique for breast pocket dissection in augmentation, but it produces thermal injury, postoperative pain, and delayed recovery. Ultrasound-assisted liposuction devices may provide a less traumatic approach for subglandular breast augmentation. The aim of this study was to evaluate the safety, feasibility, and postoperative outcomes of real-time ultrasound-guided subglandular breast augmentation using ultrasonic dissection, hypothesizing that ultrasound-assisted dissection can be performed with minimum tissue trauma, postoperative pain, and recovery time. The authors conducted a prospective cohort including healthy women (aged 21-45 years) undergoing primary subglandular breast augmentation. All patients received real-time ultrasound-guided dissection using ultrasonic devices after tumescent infiltration. Outcomes included incision size, operative time, postoperative pain measured by visual analog scale (VAS), complications, and implant characteristics. The authors included 30 patients in the cohort. The mean age was 30 years (range, 22-45 years, standard deviation [SD] 4.9). The average implant volume was 278.5 cc (range, 205-375 cc, SD 38.9), with a mean incision size of 3.0 cm (SD ±0.34). The mean surgical time was 40.2 min (range, 24-55 min, SD 7.3). Pain intensity was minimal in the immediate postoperative period, with an average baseline VAS of 1.07 and a 24 h VAS of 1.7 (SD ±0.78). No patient reported the need for extra or stronger analgesic medication, nor did they declare having taken it. Image-guided ultrasonic subglandular dissection is technically feasible, safe, and well tolerated, with minimal pain, small incisions, and no major complications in short-term follow-up. This pilot study supports the concept of ultrasonic tissue separation in aesthetic breast surgery. Larger controlled studies with longer follow-up are needed to establish its role.

**Level of Evidence**: 4 (Therapeutic) 
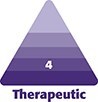

Primary breast augmentation is often perceived as a simple surgery, especially when viewed superficially. However, like any other procedure in plastic surgery, it can present a high degree of technical complexity and, in case of complications, generate important consequences, both aesthetic and functional. Achieving an optimal outcome involves facing multiple challenges, including great anatomical variability—both primary and secondary to previous surgeries—as well as a wide range of implant shapes, sizes, and projections available.^1,2^

For years, the creation of the breast pocket has relied on the use of electrocautery, a standard tool that cuts and separates tissues using heat. However, this method of dissection can reach temperatures of up to 100°C; it produces thermal injury, which may potentially worsen postoperative pain and delay recovery. Faced with these limitations, technological development has allowed the incorporation of more precise and less aggressive methods. (Video 1)^3,4^

The use of ultrasound-assisted liposuction (UAL) devices, such as HEUS (Indemex, Santo Domingo, Dominican Republic) or VASER (Solta Medical, Bothell, WA), represents a significant advance, but the indications are expanding. These technologies have been shown in liposuction as an adjuvant to extract fat more easily and with less bleeding but also to separate tissues (as observed during lipectomy following liposuction); we infer that UAL could be used in the same way to separate the mammary gland from the pectoral fascia in primary breast augmentation. Although ultrasonic devices have previously been applied to breast tissue in other contexts, this is the first report of their use for subglandular pocket dissection. By preserving the anatomical structures adjacent to the implant, inflammation may be reduced, and postcomfort may be improved with the potential for more stable results in the long term. Also, by avoiding broad tissue damage, the likelihood of requiring corrective procedures in the future may decrease.^5-7^

In this context, the use of the subglandular pocket has also been revalued, in contrast to the submuscular or dual-plane plane that was promoted for years because of its supposed advantages. The subglandular technique, when performed with careful dissection and small implants, offers benefits: it is less invasive, fast, and generates less pain by not dissecting the muscle. In addition, because it does not compromise the pectoralis major muscle, its functionality is preserved, which is especially important in cosmetic surgery, leaving open the possibility of using it later in reconstructive procedures, if necessary.^8,9^

In summary, it was hypothesized that placing the breast implant in the subglandular plane, combined with ultrasound technologies and ultrasonographic guidance, would allow adequate dissection of the subglandular pocket for breast augmentation and offer a viable alternative to placing breast implants.

## METHODS

We conducted a prospective study of women undergoing primary ultrasonic subglandular breast augmentation at the senior author's private practice between September 2024 and January 2025. Inclusion criteria were women between 21 and 45 years old with good overall health conditions. Exclusion criteria included previous breast surgery, breast ptosis or tuberous breast, or any important health problem (eg, diabetes and hypertension). Procedures were performed under local anesthesia with sedation after tumescent infiltration. The study was approved by the EMERED Hospital Ethics Committee; all patients provided written informed consent. The variables considered were age, type of anesthesia, ultrasonic platform (Heus or Vaser), incision size, postoperative pain in the first 4 and 24 h, surgical time, size and type of implant, complications, sensory alterations, type of implant projection, and visual analog scale (VAS) pain (baseline and 24 h). Minimum follow-up was considered 6 months.

Preoperative tests were requested, suitability for the procedure was confirmed, and they fasted for 8 h for their procedure on the day of surgery. Motiva implants were chosen; preferably Ergonomix implants (Motiva, Establishment Labs, Costa Rica) were used in all cases because of the ability of these implants to be placed through smaller incisions because of their rheology.^10,11^ The choice of implant was based on the measurement of the breast base, and the projection was calculated with the help of Crisalix (Lausanne, Swizerland) and the patient's choice.^12^ All implants were chosen preoperatively.

The surgery was performed under local anesthesia infiltration at the incision site with 5 cc of 2% xylocaine with epinephrine, and a puncture was immediately performed with a 15-blade scalpel where the inframammary incision would be placed. Subsequently, a solution of 500 cc of normal saline solution with 0.5 mL of epinephrine (1:1000) plus 10 mL of 7.5% ropivacaine and 20 cc of 1% xylocaine and 250 mg of tranexamic acid was prepared for infiltration with a 2.5 mm infiltration cannula. The introduction of the cannula was carried out parallel to the plane of the body, lifting the mammary gland to enter the subglandular space. To confirm that the infiltration was made in the right place, that is, in the space between the pectoral fascia and the mammary gland, it was performed under 12 to 15 MHz linear ultrasound guidance, Lumify (Phillips, the Netherlands) or Clarius L15 (Clarius Mobile Health, Vancouver, Canada) and directed in a straight line toward the paramedial line and then directed toward the subareolar region within the same plane.

In total, 180 cc of this infiltration solution was infiltrated into each breast, seeking that all the areas to be treated were infiltrated. During the introduction of the cannula, the anesthesiologist deepened the sedation so that the procedure was as comfortable as possible. Once infiltrated, we proceeded with the dissection of the subglandular plane by introducing a Heus sonotrode, at a power of 70%, or Vaser in Vaser mode at 70%, which allowed us to perform the dissection of the subglandular area. Ultrasound guidance was used throughout the procedure to confirm infiltration plane and assist in dissection. Once this dissection was performed, a 2.5 to 3 cm incision was made (which can be designed as small as the surgeon considered it), and then with electrocautery dissection is directed to the intramammary groove and find the subglandular space (Video 1). The subglandular dissection boundaries were defined by a vertical line located 1 to 1.5 cm lateral to the midline, extending to the lateral edge of the areola, and vertically limited by the implant base diameter to avoid excessive superior dissection. Once in that plane, a heart-shaped flat separator is introduced to complete the blunt dissection and separate the remaining structures, and then the implant is inserted with a funnel. Once the implant was placed, we re-approximate the inframammary fold with 2 PDS 2.0 (Ethicon, Johnson & Johnson, New Brunswick, NJ) stitches to the fascia superficialis, and then we closed the rest of the tissues and skin with Monocryl 3-0 and placed an intradermal suture with Monocryl 4-0 (Ethicon, Johnson & Johnson; Video 2). We measured the pain VAS at the discharge time and 24 h later.

### Postoperative Care

Patients were discharged after regaining ambulation, tolerating oral intake, and completing postoperative monitoring in the recovery unit. We indicated antibiotics for 7 days (cefazolin or ciprofloxacin in case of allergies) and analgesics (etoricoxib 120 mg daily, plus paracetamol 750 mg each 8 h) for 3 days. All patients were advised to rest without any specific activity restrictions, except for strenuous arm movements or exercise for 1 week. They were allowed to remain at home or engage in brief, low-effort activities outside the home and then use a bra for a month.

### Statistical Analysis

Statistical analysis was performed with descriptive, parametric, and nonparametric analysis. Correlations between variables were assessed using Pearson's correlation coefficient. Statistical significance was set at *P* < .05.

## RESULTS

**Figure 1.**
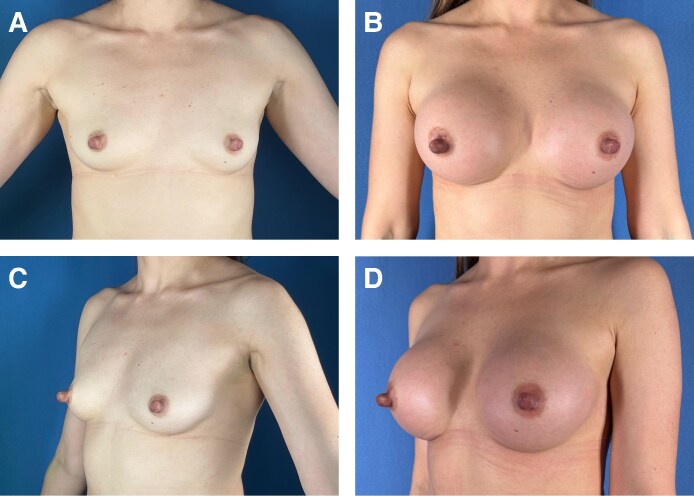
(A, C) Before and (B, D) 6 months after images of a 37-year-old female treated with ultrasonic breast augmentation using 315 cc Motiva Full ergonomix implants.

**Figure 2.**
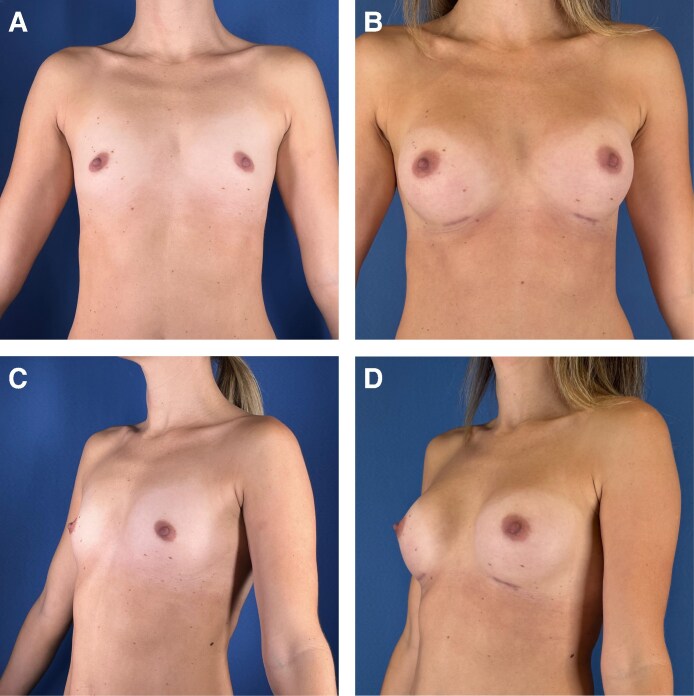
(A, C) Before and (B, D) 6 months after images of a 24-year-old female treated with ultrasonic breast augmentation using 295 cc Motiva Full ergonomix implants.

**Figure 3.**
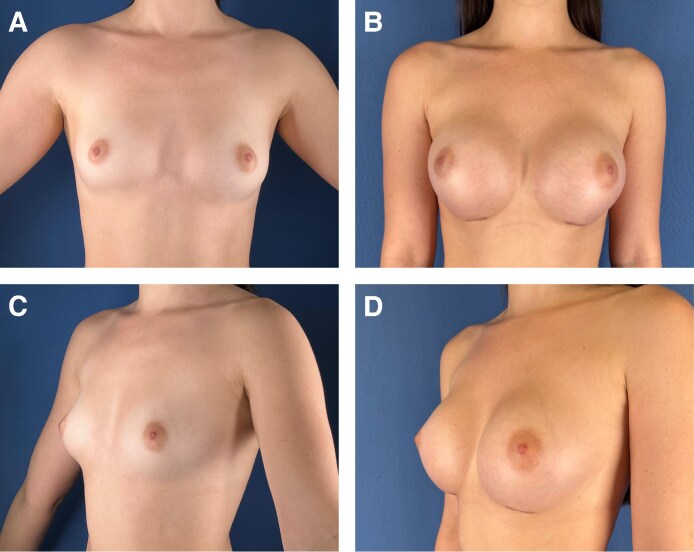
(A, C) Before and (B, D) 6 months after images of a 35-year-old female treated with ultrasonic breast augmentation using 205 cc Motiva Demi ergonomix implants.

The study began in September 2024. A total of 30 patients who underwent subglandular augmentation mammoplasty using the ultrasonic breast augmentation technique between September and January 2025 were included. They were consecutive patients. The mean age was 30 years (range, 22-45 years, standard deviation [SD] ±4.9). The average implant volume was 278.5 cc (range, 205-375 cc, SD ±38.9), with a mean incision size of 3.0 cm (range, 1.8-4.2 cm, SD ±0.34). The mean surgical time was 40.23 min (range, 24-55 min, SD ±7.3). Pain intensity was minimal on the first day, with a mean VAS score of 1.07 (range, 1-3, SD ±0.36) and on the second day of 1.7 (range, 0-2, SD ±0.78) at 24 h (Table 1). No patient reported the need for extra or stronger analgesic medication, nor did they declare having taken it. The most commonly used implant projections were full (*n* = 20), corset (*n* = 4), demi (*n* = 4), and full round (*n* = 2). Comparative analysis by implant type showed no clinically significant differences in VAS scores or surgical time. However, a strong positive correlation (Pearson) was observed between implant volume and incision size (*r* = 0.78; *P* < .001), which was the only statistically significant relationship in the multiple correlation analysis, meaning that as the implant was larger, a larger incision was required. Regarding the ultrasonic technology used, 27 patients were operated on with HEUS and 3 with VASER. No cases of seroma, bleeding, or ecchymosis were observed. Five patients (16.7%) reported a transient increase in sensitivity that subsided in the first week, and only one had a slight decrease in sensitivity in one of the breasts that was corrected at 2 weeks. The average follow-up at the time of analysis was 158 days (range, 120-210 days; SD ±31.4). Representative results of patients treated using this technique are demonstrated in Figures 1-3.

**Table 1.** Patients Included in the Cohort With Ranges, Means, and Standard Deviation

Variable	Mean	SD	Min	Max	Range
Age, years	30.33	4.93	22	45	23
Implant volume (cc)	278.5	38.93	205	375	170
Incision size (cm)	3	0.34	2.5	4	1.5
Surgical time (min)	40.23	7.31	24	55	31
Initial pain VAS	1.07	0.36	1	3	2
24 h pain VAS	1.7	0.78	1	3	2

SD, standard deviation; VAS, visual analog scale.

## DISCUSSION

For decades, breast augmentation surgery has been performed mostly by dissection of the pocket using electrocautery, an effective but inherently traumatic tool for tissues. This method, although standard, involves temperatures of up to 100°C, with well-documented side effects: internal burns, charring and vaporization of tissue planes, secondary inflammation, and increased postoperative pain. In light of these effects, the need for more tissue-friendly techniques arose in us.

In UAL, emulsification refers to the mechanical and thermal disruption of adipocytes by the action of ultrasound. Ultrasound breaks down the adipocyte's cell membrane, transforming solid fat into a liquid mixture that is easier to aspirate. However, if we decrease the energy, mechanical disruption is achieved with less thermal disruption. The findings in this study support the use of the devices used for UAL as an alternative for the creation of the subglandular pocket by performing the separation of tissues by ultrasonic waves and not by radiofrequency, allowing us for the dissection of the subglandular plane.^3^ This dissection would have the advantage of being performed under an infiltration of solution that would allow the ultrasound waves of the device to propagate and also avoid an increase in temperature so that there would be no drying or boiling that normally occurs with the use of an electrocautery that emits radiofrequency energy in tissues that are becoming desiccated and charred.^5^ This advantage of dissection and cutting of tissues with less damage, less bleeding, and less pain in the use of ultrasound compared with electrocautery has been previously demonstrated in other studies.^13-17^ Clinically, we observed results with minimal postoperative pain (initial VAS 1.07), short surgical times (mean, 40.23 min), and good functional recovery. Complemented with precise infiltration under ultrasound vision, hemostatic control with tranexamic acid, and assisted tissue expansion, this technique allowed us to have no bleeding or hematoma, no seroma, and no permanent alteration of sensation reported at the time of study closure.

**Figure 4.**
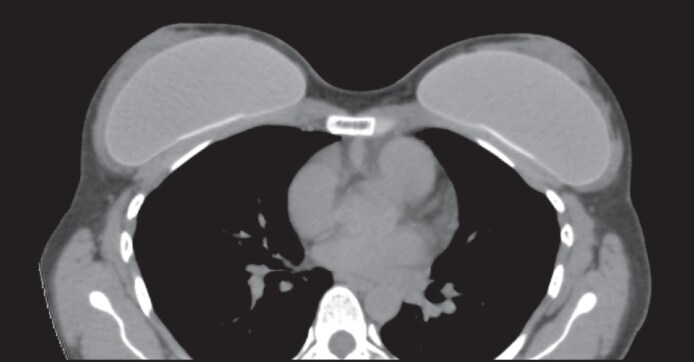
Tomography of breast in a 37-year-old female, 5 months after an ultrasonic breast implant. The breast gland can be seen above and lateral to the implant.

The technique used has been called ultrasonic breast augmentation, because it uses this technology to guide the dissection in real time with image ultrasound and also with ultrasonic devices, which allowed a dissection of the subglandular plane to be performed without the need for wide incisions and we can confirm its accuracy through endoscopic images. Ultrasonic energy allowed the tissues to be separated in a controlled manner, avoiding the thermal damage characteristic of electrocautery. This maneuver, which we called “ultrasonic tissue separation,” not only dissects but preserves key anatomical structures, such as the fascia of the pectoralis major and Cooper's ligaments and blood vessels, as occurs when used in other contexts such as liposuction in the abdomen (Video 3).^18^ This dissection of breast tissues with ultrasound has been performed previously for other purposes, demonstrating that it does not cause damage to breast tissues beyond adipocytes.^19,20^ Limited and centralized dissection for placement of implants, together with the preservation of these anatomical elements, could result in better implant stability, lower incidence of displacement, and a more natural morphology (Figure 4).

In this prospective cohort, the use of image-guided ultrasonic dissection for subglandular breast augmentation demonstrated consistent operative performance and favorable short-term outcomes. Mean operative time was 40.23 min and incision size 3.0 cm, allowing the insertion of implants up to 375 cc. Pain scores remained low throughout the early postoperative period, with a baseline VAS of 1.07 and a 24 h VAS of 1.7, and no patient required additional analgesia. Importantly, no hematomas, seromas, or major complications were observed, corresponding to a complication rate of 0% (95% CI, 0%-10%).

These findings support the feasibility and safety of ultrasonic dissection in primary breast augmentation. The ability to insert moderate-to-large implants through relatively small incisions with minimal postoperative discomfort may represent a technical advantage compared with conventional electrocautery-based dissection. Nevertheless, the absence of a control group and the limited sample size underscore the need for larger, controlled studies to validate these preliminary observations.

This study has limitations, including a small sample size, no control group to compare postoperative pain levels, and the added cost to the procedure of having this technology available. In addition, the procedure was performed by a single surgical team, which limits the generalization of the results of the technique. Another limitation is that the long-term rate of implant malposition with this ultrasonic subglandular approach remains unknown. Future controlled studies with extended follow-up will be necessary to determine whether this technique influences implant stability over time.

Future multicenter studies, with larger numbers of patients and long-term follow-up, will be needed to confirm these findings. Therefore, this study does not infer superiority of the procedure over the standard procedure, but based on the data obtained, we can infer feasibility, low pain and rapid recovery. Not all patients are suitable candidates for this procedure. This procedure should be limited to women with no previous breast surgery, excluding those with significant ptosis or with very thin soft tissues in whom the surgeon considers muscular coverage to be more appropriate.

A key advantage of this technique is that it allows for a nearly closed dissection of the breast pocket. If there were today silicone implants that could be inserted through a 0.5 cm incision, surgery would be feasible. Although there are no saline implants in Mexico, in countries where they are available, this technique could allow breast augmentations with minimal incisions. In the future, the technology may allow silicone implants to be placed through such small incisions. That is why we consider this technique to be important today, because it opens the possibility of new advances in minimally invasive breast surgery.

## CONCLUSIONS

In this prospective cohort of 30 patients undergoing subglandular breast augmentation with image-guided ultrasonic dissection, low immediate pain scores, short operative times, and no major complications were recorded during a median follow-up of 6 months. The technique allowed the implants to be inserted through small incisions, and the endoscopic inspection corroborated an adequate anatomical subglandular pocket dissection. These initial findings support the technical feasibility and short-term tolerability of the method; however, its clinical value should be confirmed by studies with a larger number of cases, prolonged follow-up and direct comparison with conventional approaches.
